# Effects of ADMA upon Gene Expression: An Insight into the Pathophysiological Significance of Raised Plasma ADMA

**DOI:** 10.1371/journal.pmed.0020264

**Published:** 2005-10-04

**Authors:** Caroline L Smith, Shelagh Anthony, Mike Hubank, James M Leiper, Patrick Vallance

**Affiliations:** **1**Centre for Clinical Pharmacology and Therapeutics, Division of Medicine, University College London, London, United Kingdom; University of UtahUnited States of America

## Abstract

**Background:**

Asymmetric dimethylarginine (ADMA) is a naturally occurring inhibitor of nitric oxide synthesis that accumulates in a wide range of diseases associated with endothelial dysfunction and enhanced atherosclerosis. Clinical studies implicate plasma ADMA as a major novel cardiovascular risk factor, but the mechanisms by which low concentrations of ADMA produce adverse effects on the cardiovascular system are unclear.

**Methods and Findings:**

We treated human coronary artery endothelial cells with pathophysiological concentrations of ADMA and assessed the effects on gene expression using U133A GeneChips (Affymetrix). Changes in several genes, including bone morphogenetic protein 2 inducible kinase *(BMP2K),* SMA-related protein 5 *(Smad5),* bone morphogenetic protein receptor 1A, and protein arginine methyltransferase 3 *(PRMT3;* also known as *HRMT1L3),* were confirmed by Northern blotting, quantitative PCR, and in some instances Western blotting analysis to detect changes in protein expression. To determine whether these changes also occurred in vivo, tissue from gene deletion mice with raised ADMA levels was examined. More than 50 genes were significantly altered in endothelial cells after treatment with pathophysiological concentrations of ADMA (2 μM). We detected specific patterns of changes that identify pathways involved in processes relevant to cardiovascular risk and pulmonary hypertension. Changes in *BMP2K* and *PRMT3* were confirmed at mRNA and protein levels, in vitro and in vivo.

**Conclusion:**

Pathophysiological concentrations of ADMA are sufficient to elicit significant changes in coronary artery endothelial cell gene expression. Changes in bone morphogenetic protein signalling, and in enzymes involved in arginine methylation, may be particularly relevant to understanding the pathophysiological significance of raised ADMA levels. This study identifies the mechanisms by which increased ADMA may contribute to common cardiovascular diseases and thereby indicates possible targets for therapies.

## Abstract 2

Pathophysiological concentrations of asymmetric dimethylarginine elicit significant changes in coronary artery endothelial cell gene expression and highlight specific molecular pathways for further investigation.

## Introduction

Asymmetric dimethylarginine (ADMA) is an endogenous inhibitor of all nitric oxide synthase (NOS) isoforms [1]. It is synthesised by the action of protein arginine methyltransferases (PRMTs), and following proteolysis, free ADMA is released into the cell cytosol and thence into plasma. Circulating concentrations of ADMA are increased in patients with renal failure [1], pulmonary hypertension, heart failure, hypercholesterolemia or a wide range of other cardiovascular risk factors [2–6]. In patients with end-stage renal failure, the plasma levels of ADMA predict mortality and cardiovascular outcome [7], and in a cohort of otherwise healthy Finnish men, those with the highest levels of ADMA had an increased risk of acute coronary events [8]. Increased circulating ADMA in pregnant women predicts an increased risk of pre-eclampsia and intrauterine growth retardation [9].

Despite these clinical observations and the increasing excitement surrounding the use of ADMA as a risk marker for vascular disease [3,7], it is still not clear whether ADMA has a causal role in pathophysiology. It has been argued that the concentration of ADMA in plasma is too low to be an effective inhibitor of NOS, and that the usual concentrations of arginine in cells should overcome any inhibitory effects of ADMA on NOS [10]. In order to determine how ADMA might exert effects on endothelial cells and produce pathology, we assessed the effects of ADMA on gene expression in human coronary endothelial cells.

## Methods

### Cell Culture

Human coronary artery endothelial cells (HCAEC) were purchased from Promocell and grown according to the manufacturer's instructions. HCAEC in 75-cm^2^ flasks at 70% confluency (passage 3 or 4) were treated for 24 h with complete media supplemented with asymmetric dimethylarginine (N^G^N^G^-dimethyl-L-arginine; ADMA; 0, 2, or 100 μM; Merck Biosciences, United Kingdom). This was repeated on three separate occasions with different batches of cells. RNA from each study was used as described below for GeneChip (Affymetrix, Santa Clara, California, United States) analysis. Our strategy for the GeneChip and subsequent analysis is outlined in Figure 1.

**Figure 1:**
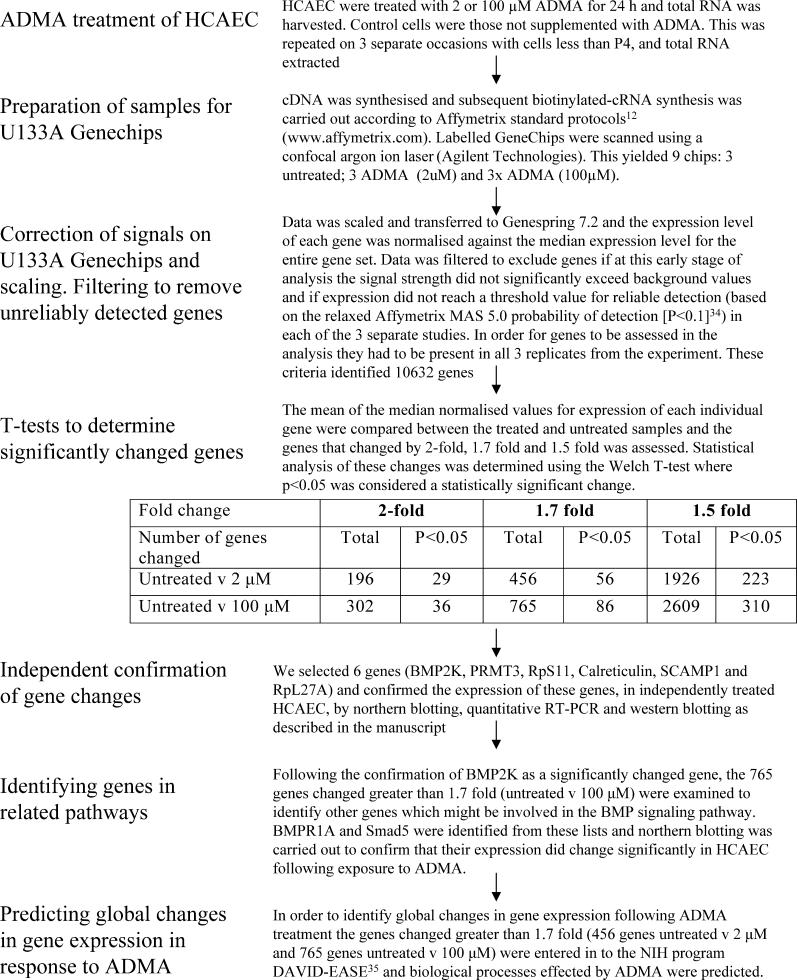
Flow Diagram Summarising the Methods Used in This Manuscript

### GeneChip Experiments

ADMA-treated HCAEC from T75 flasks were harvested in 7.5 ml of TRIzol (Invitrogen, Carlsbad, California, United States), and total RNA was extracted; cDNA and subsequent cRNA synthesis were prepared as previously described [11]. The quality of the biotin-labelled cRNA transcripts was determined using a Bioanalyser 2100 (Agilent Technologies, Palo Alto, California, United States). Purified cRNA (15 μg) was fragmented and hybridised to human U133A GeneChips according to Affymetrix standard protocols (http://www.affymetrix.com). Labelled GeneChips were scanned, using a confocal argon ion laser (Agilent Technologies).

### GeneChip Data Analysis

The U133A GeneChip contains oligonucleotides derived from approximately 22,000 human transcripts and includes control bacterial genes *bioB, bioC, bioD,* and *cre.* GeneChip data files scaled to 100 and normalised to the median prior to analysis with GeneSpring 7.2 software (Agilent; Figure 1). Genes were excluded if the signal strength did not significantly exceed background values and if expression did not reach a threshold value for reliable detection (based on the relaxed Affymetrix MAS 5.0 probability of detection (*p* ≤ 0.1; [12]) in each of the three separate studies. Finally, genes were excluded if the level of expression did not vary by more than 1.7-fold between ADMA-treated (2 or 100 μM) compared with untreated control HCAEC. The remaining genes were subjected to nonparametric Welch *t*-tests and are reported with their respective fold changes and *p*-values. The data have been submitted in a MIAME-compliant format to ArrayExpress at EBI (http://www.ebi.ac.uk/arrayexpress/).

### Determination of ADMA Levels

HCAEC cells were grown in 75-cm^2^ flasks as described above for 24 h. Methylated arginines in the conditioned medium were quantitated by HPLC as previously described [1,13].

### Cell Growth Assay

Cells were seeded into a 96-well microtitre plate at a density of 500 cells per well and grown in complete EC medium in the presence of 0, 2, or 100 μM ADMA. Cell growth was assayed (in triplicate) over a 4-d period using the CellTiter 96 Aqueous One Solution Cell Proliferation Assay (Promega, Madison, Wisconsin, United States).

### Confirmation of Gene Changes

For genes of interest selected on the initial GeneChip analysis, further experiments were undertaken, with different batches of cells, to verify changes detected by global expression profiling and using different approaches to assess mRNA or protein.

### RT-PCR

For RT-PCR cDNA was synthesised from the total RNA (approximately 1 μg) using Ready-To-Go You-Prime First-Strand Beads (Amersham Biosciences, Little Chalfont, United Kingdom) and supplemented with the 3′ gene-specific primers with *β-actin* as a control. RT-PCR used the primer sequences (Table 1) with *β-actin* as a control.

**Table 1:**
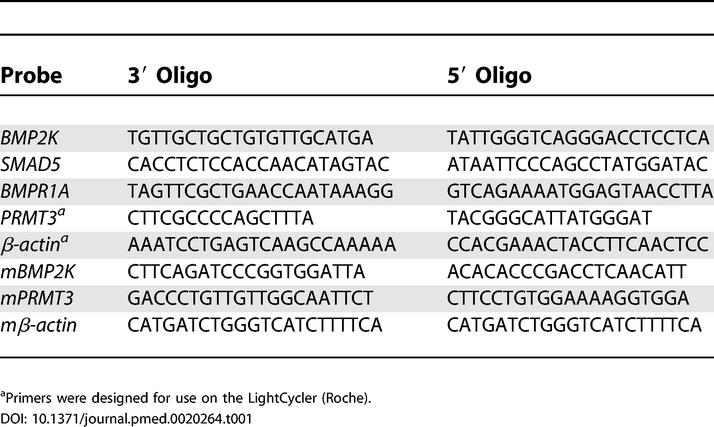
Primer Sequences for Q-PCR and RT-PCR

The PCR products were separated in 1% agarose gel, and the intensity of the bands was measured; each sample was corrected for *β-actin*. PCR products used for Northern blotting were excised and purified from agarose gel using Qiaex II (Qiagen, Valencia, California, United States).

### Quantitative-PCR

Quantitative PCR (Q-PCR) was carried out using a LightCycler (Roche, Alameda, California, United States) and LightCycler software version 3, LightCycler run 4.24. All reagents required for PCR (excluding cDNA and primers) were included in the LightCycler FastStart DNA Master SYBR Green 1 kit (Roche). Reverse transcription was performed as described above for RT-PCR. The PCR cycle settings were 95 °C for 5 min, followed by 45 cycles of 95 °C for 5 s, 58 °C for 10 s (60 °C for *β-actin*), 72 °C for 40 s, and 77 °C (82 °C for *β-actin*), where fluorescence was measured at the end of each cycle and is gene-specific. Standard curves were constructed for *PRMT3* as described by the manufacturer's instructions and compared to the reference gene *β*-*actin.*

### Northern Blotting

HCAEC were grown to 70% confluency in 6-well plates and treated with 0, 2, and 100 μM ADMA, 100 μM L-N^5^-(1-Iminoethyl)ornithine (L-NIO), or 100 μM symmetric dimethylarginine (SDMA) for 24 h; total RNA was extracted using TRIzol, and RNA was separated by gel electrophoresis and blotted onto Hybond N^+^ (Amersham Biosciences). The ribosomal protein S11 (*RpS11*), ribosomal protein L-27 (*RpL-27*), Calreticulin, and secretory carrier membrane protein 1 *(SCAMP1)* probes were generated by end-labelling 5′ oligonucleotides (Table 2) using T4 polynucleotide kinase.

**Table 2:**
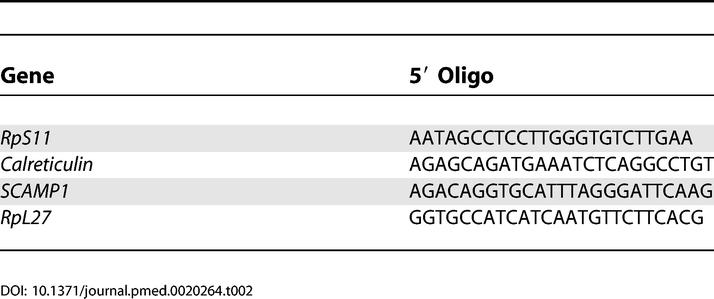
Oligonucleotide Sequences for 5′-End-Labelling Northern Blotting Experiments

The PCR products from RT-PCR reaction for β*-actin, Smad5,* bone morphogenetic protein receptor 1A *(BMPR1A),* and murine β-actin (*mβ-actin*); murine bone morphogenetic protein 2 inducible kinase *(mBMP2K),* and murine protein arginine methyltransferase 3 *(mPRMT3)* were purified and labelled with Redivue deoxycytidine 5′-[α-^32^P]-triphosphate (Amersham Biosciences), using a random primed DNA labelling kit (Roche).

### Western Blotting

HCAEC were treated with either 0, 2, or 100 μM ADMA for 24 h in 6-well plates and harvested in lysis buffer as described previously [11], the protein concentrations of the lysates were determined by protein assay (Bio-Rad, Hercules, California, United States), and cell lysates were resolved by 12% SDS polyacryamide gel electrophoresis with equal amounts of protein loaded into each lane. Anti-PRMT3 (Upstate Biotechnology, Charlottesville, Virginia, United States) and Anti-BMP2K (Orbigen, San Diego, California, United States) were used with anti-rabbit secondary antibody coupled to horseradish peroxidase and detected with the ECL+ detection system (Amersham Pharmacia, Piscataway, New Jersey, United States). Densitometry of the bands was determined, and results are shown as the mean densitometry, where *n* = 4 with inset of a typical Western blot.

### Pathway Mapping and Gene Ontology Analysis

In order to determine whether ADMA had affected expression of genes in pathways related to the genes identified on the initial analysis, lists of genes that were changed more than 1.7-fold compared to control (irrespective of *p*-value) were examined using Gene Ontology (Affymetrix) data mining for biological process (at level 3), and Expression Analysis Systematic Explorer (EASE) biological theme analysis were conducted online at http://david.niaid.nih.gov using DAVID [14]. DAVID-EASE [15] generates an EASE score predicting the likelihood of genes mapping to specific biological processes (determined by Gene Ontology consortium) from a given list of changed genes, therefore enabling global themes in gene expression following ADMA treatment to be identified [15].

### Dimethylarginine Dimethylaminohydrolase 1 Gene Deletion Mice

ADMA is metabolised to citrulline and dimethylamine by the action of dimethylarginine dimethylaminohydrolase (DDAH). We have created knockout mice that lack DDAH1. DDAH1 heterozygous knockout mice (details to be published elsewhere) have approximately 2-fold higher plasma ADMA levels compared to wild-type litter-mates and thus provide an excellent model to test the effects of moderately raised ADMA levels in vivo. Northern blotting was carried out using RNA extracted from the brain, heart, and kidney of 12–14-wk-old DDAH1 heterozygous knockout and wild-type litter-mates with probes for *mBMP2K* and *mPRMT3,* and results are expressed relative to *mβ-actin.*

### Statistical Analyses

Q-PCR, Northern blot, and Western blot densitometry data for the treated HCAEC was analysed by one-way analysis of variance (ANOVA) coupled to Bonferroni posttest, and the Bonferroni posttest *p*-values are reported. The Northern blots from the DDAH1 gene deletion mice were compared with unpaired *t*-test and the *p*-values are reported.

## Results

### Changes in HCAEC Gene Expression in Response to ADMA on U133A GeneChips

A total of 979 genes changed in expression between ADMA-treated and control cells. Following the Welch *t*-test with a cutoff of *p* < 0.05, 56 genes were identified as having shown a statistically significant change between the untreated and 2-μM ADMA-treated cells, and 86 genes changed between the untreated and 100-μM ADMA greater than 1.7-fold; 11 genes showed statistically significant changes at both concentrations of ADMA compared to untreated cells (Figure 2A; Tables 3 and 4).

**Figure 2:**
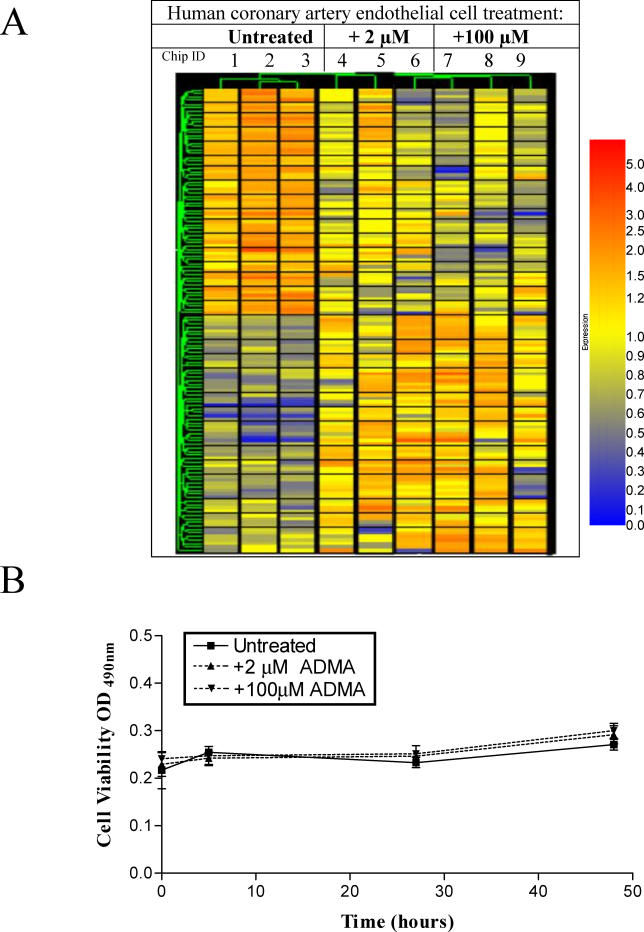
Endothelial Gene Expression Changes in Response to ADMA (A) Changes in HCAEC gene expression in response to ADMA. Hierarchical clustering of 131 genes significantly up- or downregulated (*p* < 0.05, Welch *t*-test) by greater than 1.7-fold on the U133A GeneChips in response to 2 μM ADMA (56 genes) or 100 μM ADMA (86 genes) compared with untreated cells with 11 genes changing with both concentrations of ADMA. Individual arrays are shown for each treatment group with blue representing low expression, and red high expression on the scale bar. (B) Treatment of HCAEC with either 2 μM or 100 μM ADMA had no significant effect on cell viability; *n* = 6.

**Table 3:**
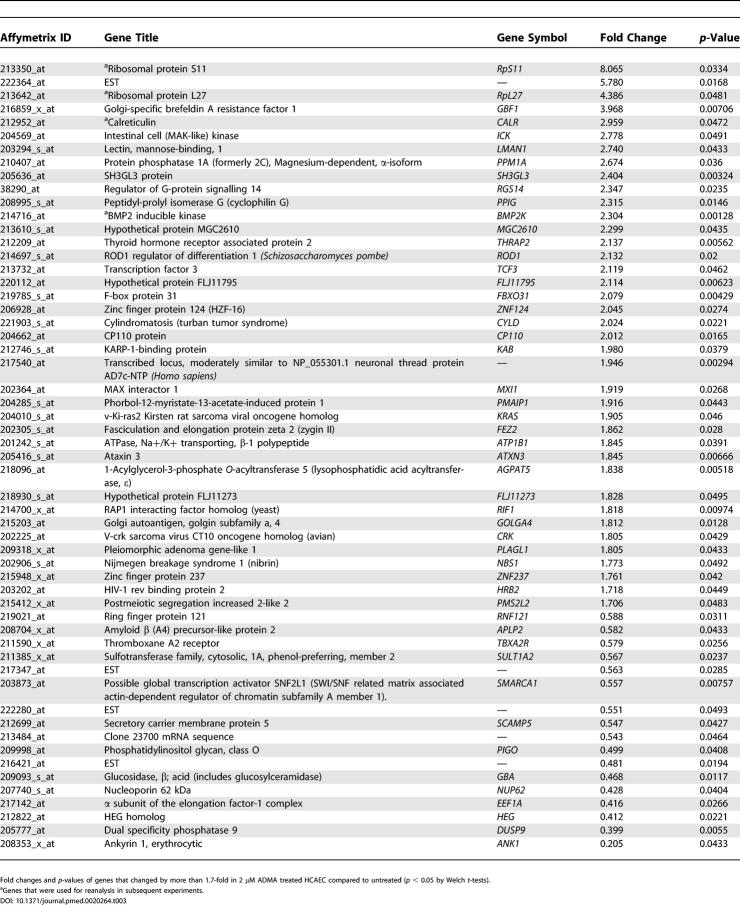
Genes That Changed by More Than 1.7-Fold in 2 μM ADMA Treated HCAEC Compared to Untreated

**Table 4:**
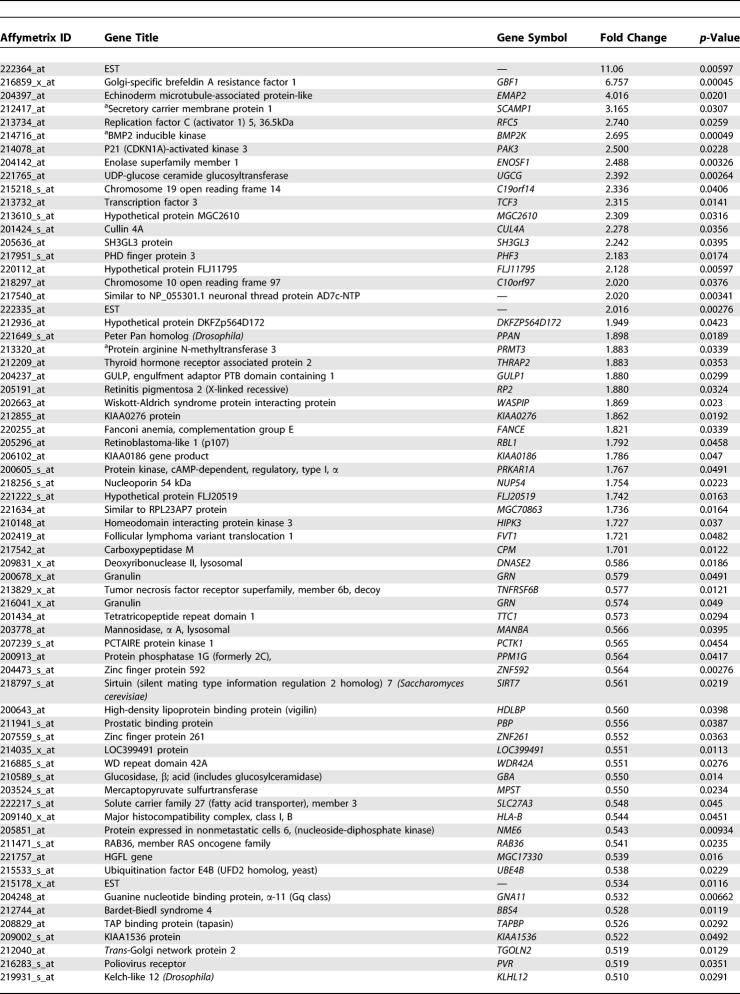
Genes that Changed by More than 1.7-Fold in 100 μM ADMA-Treated HCAEC Compared to Untreated

**Table 4:**
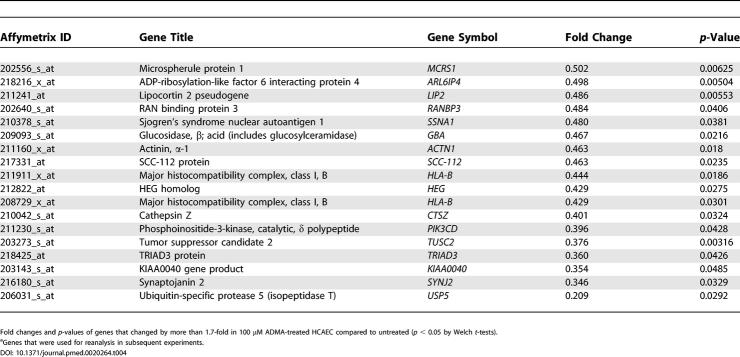
Continued

### Effects of ADMA upon HCAEC Viability

Basal levels of ADMA and SDMA in HCAEC media were 0.17 ± 0.01 μM and 0.22 ± 0.02 μM, respectively, and arginine levels exceeded 300 μM. No changes were observed in HCAEC viability over 72 h in the presence of either 2 or 100 μM ADMA (Figure 2B).

### Confirming Transcriptional Changes

To determine the reliability of changes identified by GeneChip analysis, four genes were selected from those that showed a statistically significant change in either the 2 or 100 μM sample compared to control (Figure 3A). These were *SCAMP1, Calreticulin, (RpL27)* and *RpS11.* In studies on a different batch of HCAEC, Northern blotting confirmed that expression of these genes changed in response to ADMA (Figure 3B).

**Figure 3:**
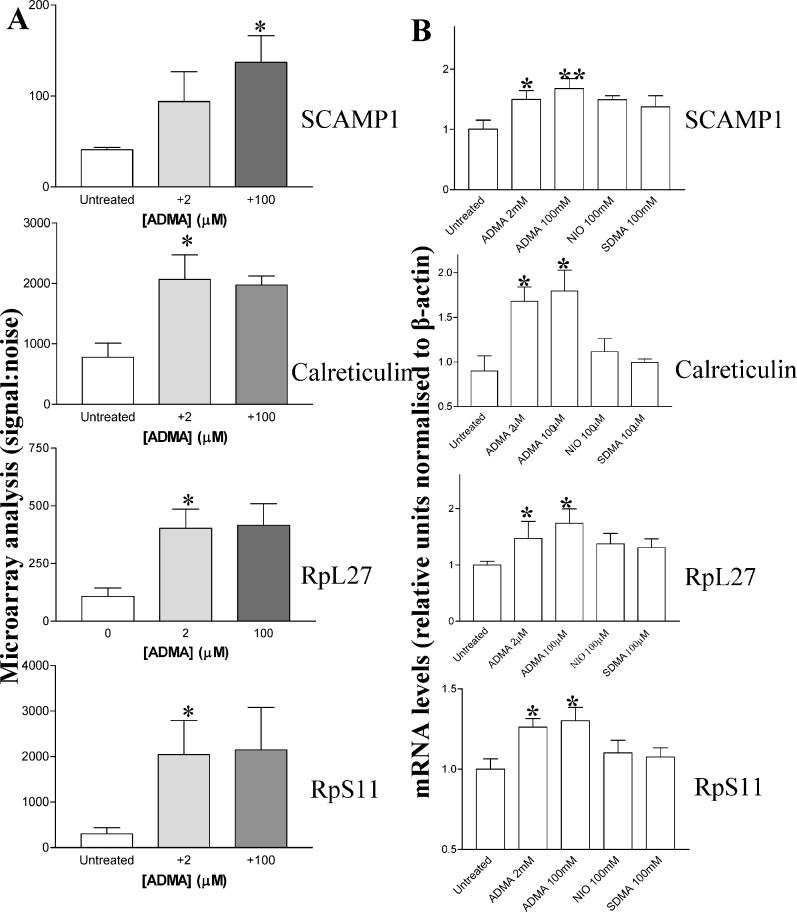
Confirmation of Gene Expression Changed by GeneChip Analysis (A) *SCAMP1; Calreticulin,* ribosomal protein L-27 *(RpL27),* and *RpS11* signal-to-noise ratios derived from U133A GeneChip analysis where *n* = 3 (untreated versus 100 μM: *SCAMP1* changed 3.16-fold, *p* = 0.031; and untreated versus 2 μM: *Calreticulin* changed 2.96-fold, *p* = 0.047; *RpS11,* 8.065-fold, *p* = 0.033; *RpL27,* 4.39-fold, *p* = 0.048). (B) *SCAMP1; Calreticulin, RpL27*, and *RpS11* mRNA levels are elevated by ADMA (2 and 100 μM; *p < 0.05 and **p < 0.01). SDMA (100 μM) and L-NIO (100 μM) did not elicit changes in gene expression as shown by Northern blotting, where mRNA was corrected for differences in β-actin mRNA expression.

In order to elucidate the mechanism of ADMA action, HCAEC were also treated with the potent NOS inhibitor L-NIO and SDMA, which is not a NOS inhibitor or DDAH substrate, but is a naturally occurring methylarginine that competes with arginine for the cationic amino acid transporter [10,16]. Interestingly neither SDMA nor L-NIO elicited significant changes in the expression of *RpS11, RpL27, SCAMP1,* or *Calreticulin* (Figure 3B).

### Genes of Specific Interest Identified by GeneChip

#### Protein arginine methyltransferase

*PRMT3* was selected as a gene of interest, because of its involvement in ADMA synthesis [17]. It changed 1.8-fold on the GeneChip untreated versus 100 μM groups (*p* = 0.0339; Figure 4A). In a separate series of follow-up studies, HCAEC were treated with ADMA, and *PRMT3* gene expression was determined by Q-PCR. *PRMT3* mRNA levels increased following either low- or high-dose ADMA treatment (Figure 4B untreated versus 2 μM, *p* < 0.05; and untreated versus 100 μM, *p* < 0.01, *n* = 6). PRMT3 protein expression also increased in response to ADMA treatment (Figure 4C; untreated versus 2 μM; and untreated versus 100 μM, *p* < 0.05, *n* = 4). When HCAEC were treated for 24 h with 2 μM SDMA or L-NIO, neither treatment significantly affected *PRMT3* expression, whereas 100 μM SDMA or L-NIO caused a small increase in *PRMT3* expression (Figure 4B; *n* = 6).

**Figure 4:**
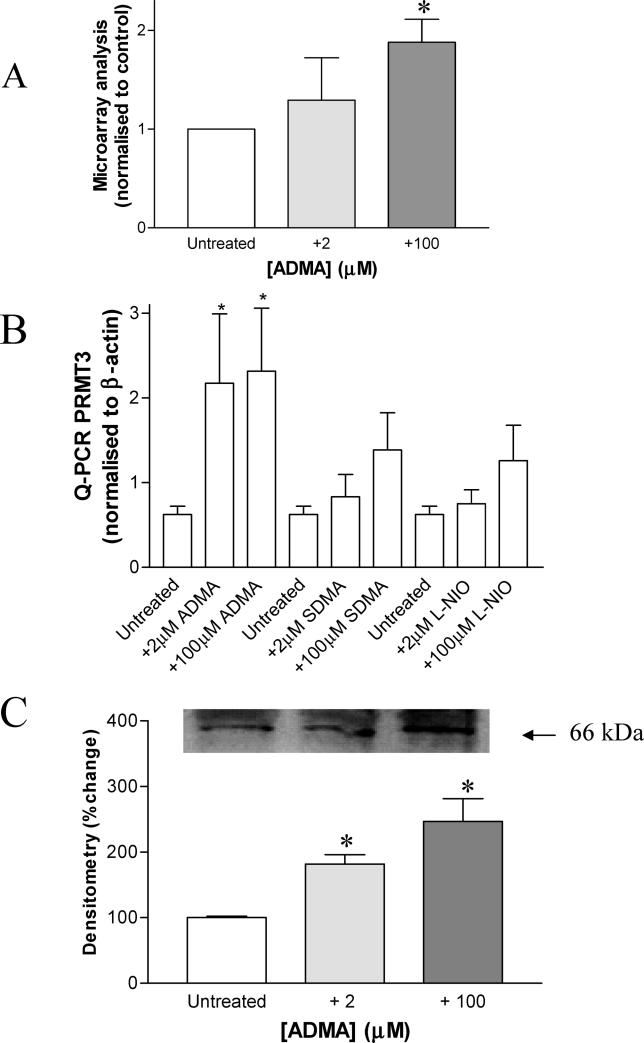
ADMA Alters PRMT3 Gene Expression and Protein Levels (A) *PRMT3* levels are changed by ADMA on U133A GeneChips where *n* = 3 (untreated versus 100 μM: 1.88-fold increase, *p* = 0.034). (B) *PRMT3* mRNA is changed in HCAEC following 24-h exposure to ADMA (2 and 100 μM) but not SDMA (100 μM) or L-NIO (100 μM), measured by Q-PCR (*p < 0.05 and **p < 0.01, where *n* = 8). (C) PRMT3 protein levels are increased in HCAEC following 24-h treatment with ADMA (2 and 100 μM) as determined by Western blotting, using a commercially available PRMT3 antibody (Upstate Biotechnology), where equal amounts of protein were loaded in each lane. Densitometry of the PRMT3 was carried for each of the blots from four separate experiments. ADMA (2 and 100 μM) treatment for 24 h significantly increased the levels of PRMT3 (*p < 0.05). The inset blot is a representative from four separate experiments.

#### Bone morphogenetic protein 2 inducible kinase

We identified that bone morphogenetic protein 2 inducible kinase *(BMP2K)* changed on the GeneChip in response to ADMA (2 μM and 100 μM ADMA increased expression by 2.304-fold [*p* = 0.00128] and 2.695-fold [*p* < 0.001], respectively; Figure 5A). In a separate series of experiments on a different batch of HCAEC this increase in *BMP2K* expression was confirmed by RT-PCR (data not shown). Western blotting also revealed that BMP2K protein levels were increased in response to ADMA (Figure 5B); densitometry of these blots indicated that there was a significant increase (untreated versus 2 μM, *p* < 0.05; and untreated versus 100 μM, *p* < 0.01, *n* = 4).

**Figure 5:**
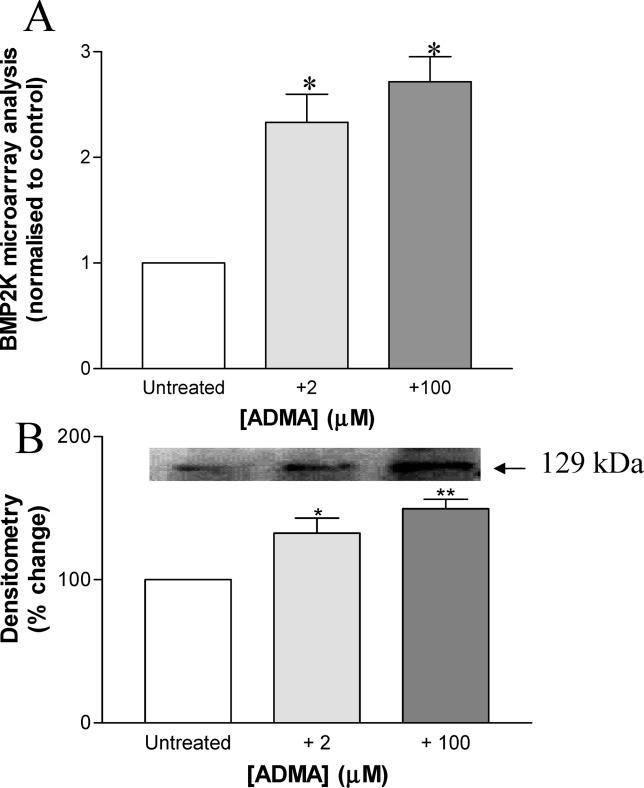
ADMA Alters BMP2K Gene Expression and Protein Levels (A) *BMP2K* is increased more than 2-fold in HCAEC following 24-h ADMA treatment (2 μM and 100 μM ADMA increased expression by 2.304-fold [*p* = 0.00128] and 2.695-fold [*p* < 0.001] respectively). (B) BMP2K protein levels are increased in HCAEC following 24-h treatment with ADMA (2 and 100 μM) as determined by Western blotting, using a commercially available BMP2K antibody (Orbigen). Densitometry of the BMP2K band was carried for each of the blots from four separate experiments (untreated versus 2 μM: *p* < 0.05; and untreated versus 100 μM: *p* < 0.01, *n* = 4). The inset blot is representative of four separate experiments, where equal amounts of protein were loaded in each lane.

### Identification of Genes Involved in Bone Morphogenetic Protein Signalling

Having confirmed changes in *BMP2K* expression, the total list of 765 genes that changed greater than 1.7-fold in response to 100 μM ADMA, was reexamined to identify additional genes in the bone morphogenetic protein (BMP) signalling pathway affected by ADMA. This analysis identified *Smad5* and *BMPR1A* (Figures 6 and 7). In a separate set of studies Northern blotting confirmed that mRNA was increased in HCAEC after 24 h by either 2 or 100 μM ADMA for *Smad5* and *BMPR1A* (*Smad5* untreated versus 2 μM and untreated versus 100 μM; *p* < 0.05, *n* = 4; Figure 6A; and *BMPR1A* untreated versus 2 μM, *p* < 0.05 and untreated versus 100 μM; *p* < 0.01, *n* = 4; Figure 6B).

**Figure 7:**
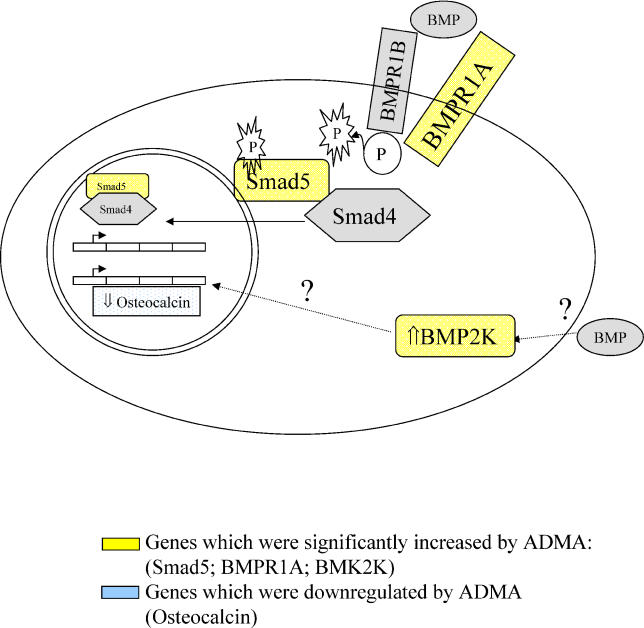
Involvement of ADMA-Affected Genes in the BMP Signalling Pathway Squares in yellow show genes that increased significantly on the GeneChip, whilst squares in blue show genes decreased by ADMA compared to untreated HCAEC, and “?” represents an unknown mechanism of action.

**Figure 6:**
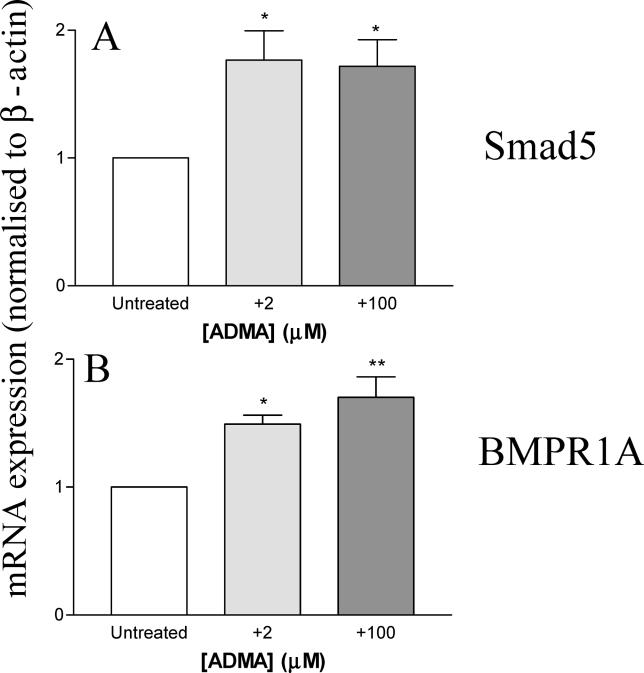
Genes Involved in BMP Signalling are Altered by ADMA *Smad5* (A) and *BMPR1A* (B) are elevated in HCAEC following 24-h treatment with ADMA (2 and 100 μM), as shown by Northern blotting where mRNA was corrected for differences in *β-actin* mRNA expression (*p < 0.05; ** *p* < 0.01, where *n* = 4).

### Identification of Global Changes in Gene Expression

All genes that changed by more than 1.7-fold (irrespective of *p*-value) were entered into DAVID-EASE. EASE probability scores were generated based upon the number of genes for each biological process altered in response to ADMA (Tables 5 and 6), where these biological processes were defined by enriched Gene Ontology categories. These gene lists indicated that ADMA affects genes involved in metabolism, RNA splicing, transcription, and cell cycle regulation.

**Table 5:**
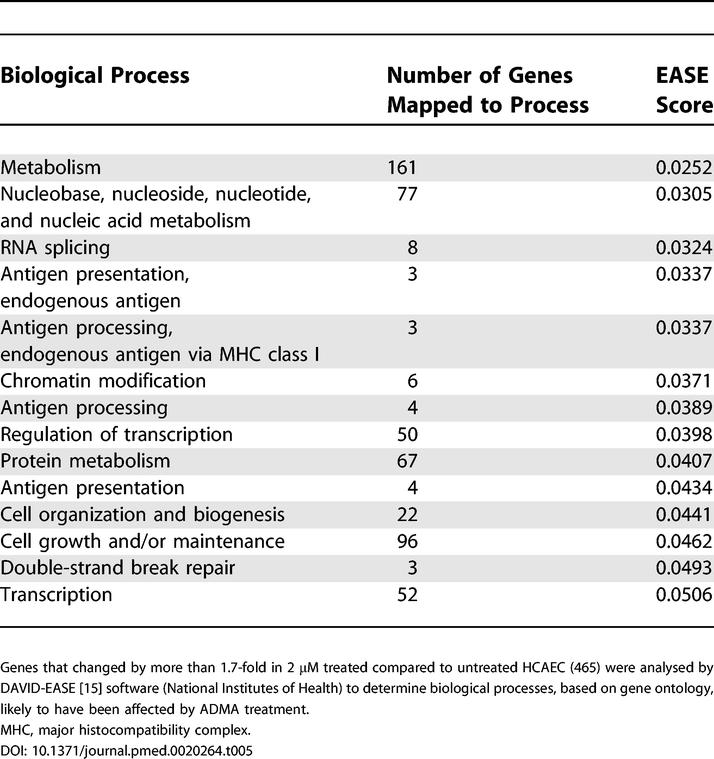
Identification of Global Changes in Gene Expression in HCAEC Following Treatment with 2 μM ADMA

**Table 6:**
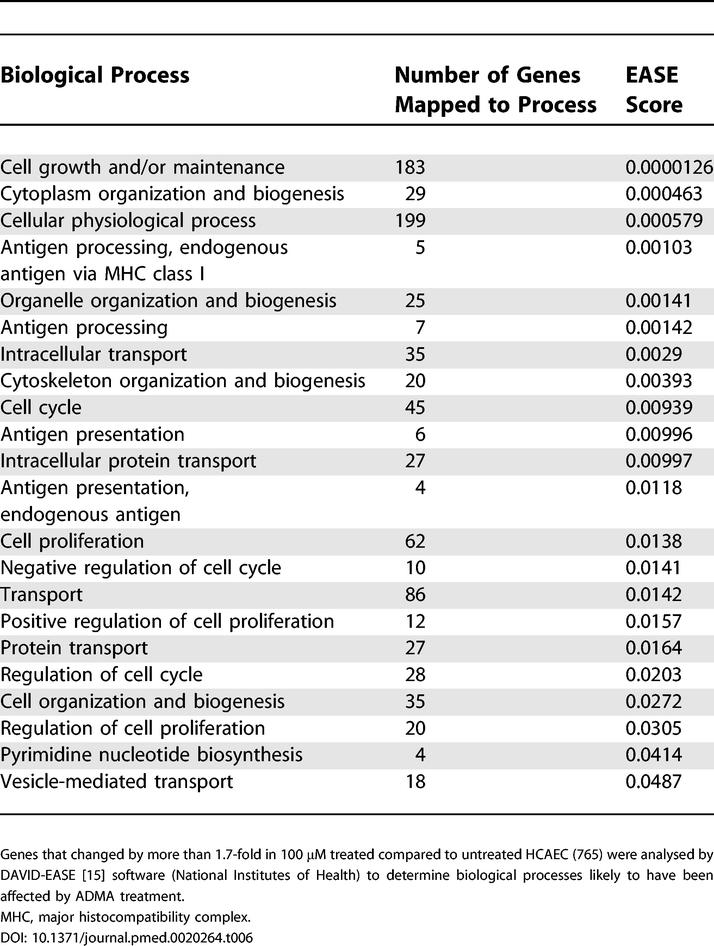
Identification of Global Changes in Gene Expression in HCAEC Following Treatment with 100 μM ADMA

### Gene Deletion Mice

To examine whether the effects observed in the cell culture model were relevant to the in vivo situation, we determined the expression level of certain genes in DDAH heterozygous knockout mice that have 2-fold elevation in plasma ADMA levels. Total RNA from DDAH1 heterozygous knockout was probed for *mBMP2K* and *mPRMT3* by Northern blotting and corrected for *mβ-actin* expression (Figure 8). Levels of *mBMP2K* for brain, heart, and kidney, respectively, were 42 ± 13.8% (*p* = 0.047), 33.3 ± 18.6% (*p* = 0.038), 74.0 ± 21.4% (*p* = 0.007), higher in DDAH1 heterozygous mice (*n* = 9) compared with wild-type litter-mates (*n* = 5). A similar trend was seen for the expression of *mPRMT3* (data not shown).

**Figure 8:**
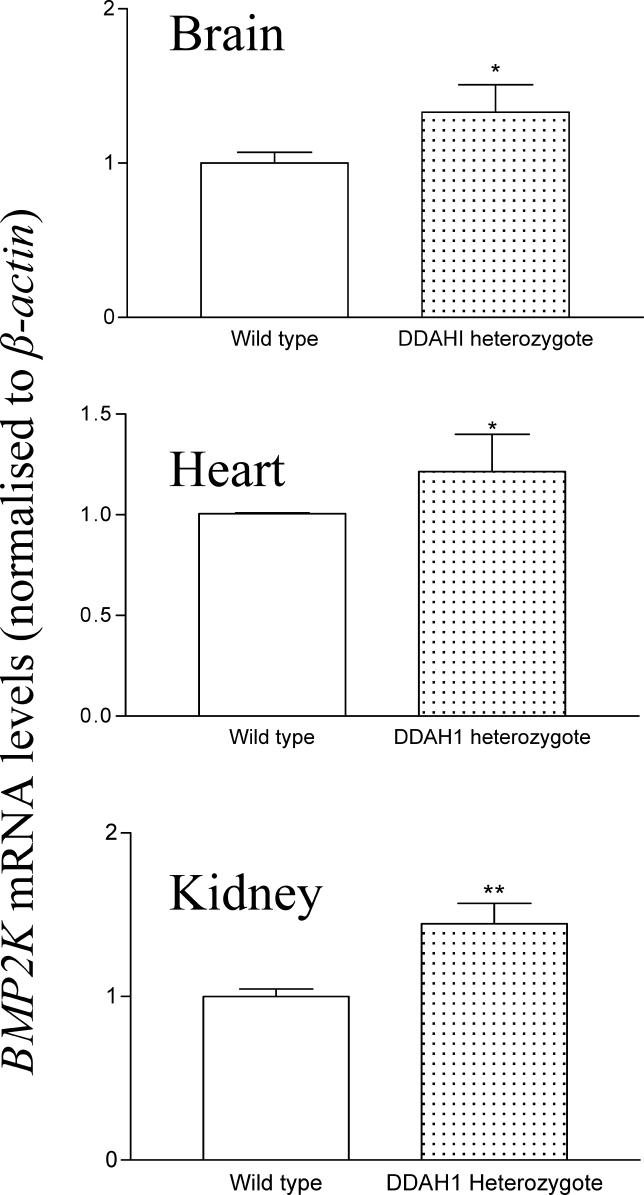
BMP2K Is Increased in DDAH1 Gene Deletion Mice Expression of *BMP2K* mRNA from brain, heart, and kidney is increased in 12-wk DDAH1 heterozygous knockout mice compared to wild-type litter-mates (*p* = 0.0473, *p* = 0.0379, and *p* = 0.0070, respectively); mRNA was corrected for differences in *β-actin* mRNA expression, where *n* = 5 wild type, and *n* = 9 DDAH heterozygous.

## Discussion

ADMA is an endogenous inhibitor of NOSs [1] and there is an association between increased plasma levels of ADMA and renal disease [1], pulmonary hypertension [5], preeclampsia [9], and the progression of atherosclerosis [18,19]. Whilst the concentration of ADMA in plasma of healthy adults varies between 0.4 and 1 μM, it may increase to 1.45–4.0 μM with certain diseases, and this increase is thought to be causally involved in pathophysiology [1,6,7,9,20]. In the present study we detected substantial changes in gene expression in HCAEC after 24 h of exposure to concentrations of ADMA similar to those reported in pathophysiological states. Furthermore, we identified specific pathways of gene activation that give insight into the mechanisms by which ADMA may contribute to disease. Surprisingly, some of these changes appear to be independent of blockade of the L-arginine:nitric oxide (NO) pathway.

### Low Concentrations of ADMA Alter Gene Expression

Acute administration of ADMA to healthy individuals elicits a transient fall in heart rate and cardiac output and increases blood pressure [2], but little is known of the potential longer-term effects of raised ADMA. Zoccali et al. reported that a 1-μM increment in ADMA above the upper limits for healthy individuals was associated with increased risk of cardiovascular mortality [7], and levels around 2 μM seem to be associated with a number of diverse cardiovascular pathologies [3,8]. In the current study, HCAEC were treated with 2 or 100 μM ADMA. We repeated GeneChip analysis in three separate studies and observed reproducible changes in the expression of a subset of genes in response to low- and high -dose ADMA. Because there is always a possibility of false positives being identified on arrays, we tested the reproducibility of the GeneChip approach by selecting four genes significantly upregulated by ADMA treatment. In a separate series of studies we confirmed an increase in mRNA levels by Northern blotting at both concentrations of ADMA. Thus it is clear that pathophysiological concentrations of ADMA (2 μM) affect endothelial cell gene expression even in the presence of very high arginine concentrations (>300 μM). Endogenous ADMA is metabolised by DDAH, and DDAH activity is the major determinant of plasma ADMA concentrations [2]. To determine whether the effects we saw in vitro would be reproduced in vivo we examined DDAH1 knockout mice. At least one of the gene changes we saw in vitro also occurs in vivo since DDAH1 heterozygous knockout mice have increased concentrations of ADMA in plasma and showed upregulation of *BMP2K* in several tissues.

ADMA inhibits NOSs with an IC_50_ of about 5 μM, the precise potency depending on the prevailing concentration of arginine [21]. It is known that adding endogenous NO to endothelial cells alters gene expression [22,23] and that inhibitors of endogenous NO generation can alter expression of specific genes, at least under conditions of endothelial cell activation with cytokines. To determine whether the effects we observed could be accounted for by inhibition of NOS, we treated cells with a highly potent NOS inhibitor (L-NIO) and with SDMA, an endogenous dimethylarginine that has no effect on NOS but which can block arginine transport [10,16]. Neither SDMA nor L-NIO replicated the effects of ADMA on gene expression. We have not undertaken a full GeneChip analysis of responses to L-NIO, so we do not know how much overlap there would be between the effects of L-NIO and ADMA, but of those genes examined we saw a discordance between responses to the two inhibitors. This raises the intriguing possibility that some of the actions of ADMA may be independent of effects of NO, possibly due to other actions such as the ability of ADMA to increase superoxide generation [24,25]. Other authors have reported differences in efficacy of ADMA in cell culture systems compared to other NOS inhibitors that have more potent effects on isolated NOS [26], and further studies will be required to identify to what extent NO-independent effects contribute to the overall action of ADMA. Interestingly, a NOS-independent effect of ADMA on angiotensin-converting enzyme has recently been suggested [25]. NOS-independent effects of ADMA may explain why, in some situations where ADMA concentrations are raised, L-arginine does not restore normal endothelial function [27] and why ADMA can exert effects, even in the presence of high endogenous arginine concentrations.

### Patterns of Gene Change

Mapping genes changed by ADMA to identify global changes in biological processes [15], indicated that ADMA treatment may have significant effects on genes involved in cell cycle regulation, cell proliferation, DNA repair, transcriptional regulation, and metabolism. The full biological significance of the range of genes affected is not yet known, but our data demonstrate the potential for elevated ADMA to affect endothelial (and likely other cellular) function in disease. In the present study, we focussed on two pathways of potential importance—BMP pathways and PRMTs.

### BMP Pathways and ADMA

Analysis of the U133A GeneChips revealed that *BMP2K* was induced more than 2-fold in response to either 2 or 100 μM ADMA. The increase in gene expression was mirrored by an increase in BMP2K protein, and the effect was also seen in our high-ADMA mouse model. By relaxing parameters (to exclude false negatives) a search for other genes involved in BMP signalling revealed that *Smad5* and *BMPR1A* were also amongst the transcripts increased by GeneChip analysis, and these changes were confirmed by Northern blotting. The finding of changes in the BMP signalling pathway is important since the ADMA/DDAH pathway seems to be involved in animal models of pulmonary hypertension [28,29], and mutations in the BMP receptor 2 (BMPR2) are associated with familial pulmonary hypertension in humans [30].

The changes in BMP pathways may also be important in understanding some of the effects of renal failure. ADMA accumulates in renal failure and fulfils many of the criteria of a uraemic toxin [1,18]. In addition to effects on cardiovascular risk, ADMA may contribute to renal osteodystrophy, a process in which BMPs have been implicated [31]. Indeed an earlier study that showed that ADMA reduces osteoblast differentiation and decreases osteocalcin expression [32]. The present study confirms osteocalcin as a gene downregulated by ADMA, and since activation of *BMP2K* attenuates osteocalcin expression and reduces osteoblast differentiation [33], it is possible that the effects of ADMA on osteocalcin may be secondary to induction of *BMP2K* (Figure 7). Whatever the mechanisms, identification of a link between ADMA and BMP pathways may be relevant to the increased vascular calcification seen in renal disease [31].

### ADMA and Arginine Methylation

We observed that *PRMT3* gene expression was elevated following exposure to ADMA; this was confirmed by Q-PCR, and PRMT3 protein expression also increased. This is the first report that ADMA may alter the expression of enzymes involved in its own synthesis. There are presently five known PRMTs that asymmetrically methylate arginine residues and two (PRMT5 and PRMT7) that symmetrically methylate arginine residues. PRMT3 has a wide tissue distribution, is expressed in highly vascular tissues, including heart and lung [17], and expression may be increased by oxidised-LDL [34]. Our observations indicate that ADMA can induce a similar increase in PRMT3 expression.

The roles of methylation of arginine residues in proteins are not yet well defined, but studies of PRMT3 in fission yeast have shown that it associates with proteins involved in the translational machinery and that the S2 ribosomal protein, a component of the yeast 40S ribosome, is a specific substrate for PRMT3 [35]. PRMT3 is the only PRMT known to interact with the translational machinery, and it is interesting that we have found several genes, including ribosomal proteins *RpS11* and *RpL27,* involved in translational control, that were also altered in response to ADMA treatment. Amongst the genes changed greater than 1.7-fold in response to ADMA was methionine adenosyltransferase II α, which catalyses the production of S-adenosylmethionine, the methyl donor for the PRMT reaction [36]. The role of ADMA in regulating arginine methylation in protein deserves further study.

### Summary

Increased circulating concentrations of ADMA have been reported in cardiovascular and other disorders, and intracellular concentrations may vary independently of circulating levels. In the present study we have demonstrated that relatively small changes in the concentration of ADMA affect gene expression in endothelial cells. Identification of pathways regulated by ADMA may aid our understanding of how ADMA contributes to a wide range of pathologies. Two pathways of specific interest have been identified—BMP signalling and enzymes involved in arginine methylation. The effects on BMP signalling may be particularly important in renal disease and in the link between raised ADMA and pulmonary hypertension.

## Supporting Information

### Accession Numbers

The microarray data have been loaded into the EBI MIAMExpress database (http://www.ebi.ac.uk/miamexpress/) and have been assigned the accession number E-MEXP-377.

**Patient Summary**

**Background**

Diseases of the circulation system are common and cause many deaths. Medical conditions associated with damage to the blood vessels include heart failure, high blood pressure, stroke, and kidney failure. The lining of the blood vessels plays an active role in maintaining their health. A substance called asymmetric dimethylarginine (ADMA) is found naturally in the vessel lining, both in healthy people and in people with vascular disease, but in the latter it is present at higher levels. Thus raised ADMA may be a marker of vascular disease. This means it could be used to help identify people with a circulation problem. However, it has not been clear whether ADMA actually causes any damage, i.e., whether it is more than just a marker.

**What Did the Researchers Do and Find?**

The researchers are trying to find out whether elevated ADMA levels can cause vascular disease. In this study, they treated cells from blood vessel linings with levels of ADMA equal to those found in people with vascular disease and measured how gene activity changed in response. They found that a number of genes were more active when the cells were exposed to the elevated ADMA levels. Some of these were interesting because other studies suggest that they might be involved in lung, heart, and kidney disease.

**What Do the Results Mean for Patients?**

This area of research is still at an exploratory stage. Additional studies need to examine which function (if any) the genes that respond to elevated ADMA levels play in vascular disease. If they do play active roles, drugs that inhibit them might help to prevent or treat vascular disease.

**Where Can I Get More Information?**

For general information on cardiovascular disease see information provided by the following organisations.

The British Heart Foundation:

http://www.bhf.org.uk/hearthealth/index_home.asp?SecID=1

The American Academy of Family Physicians:

http://familydoctor.org/292.xml

The National Heart, Lung, and Blood Institute:

http://www.nhlbi.nih.gov/health/public/heart/index.htm

New York Online Access to Health:

http://www.noah-health.org/en/blood/vascular/index.html

University College London:

http://www.ucl.ac.uk/medicine/clinical-pharmaco/research

## Abbreviations

ADMA: asymmetric dimethylarginine
BMP: bone morphogenetic protein
*BMPR1A*: bone morphogenetic protein receptor 1A
*BMP2K*: bone morphogenetic protein 2 inducible kinase
DDAH: dimethylarginine dimethylaminohydrolase
HCAEC: human coronary artery endothelial cells
L-NIO: L-N5-(1-Iminoethyl)ornithine
*mβ-actin*: murine β-actin
*mBMP2K*: murine bone morphogenetic protein 2 inducible kinase
*mPRMT3*: murine protein arginine methyltransferase 3
NO: nitric oxide
NOS: nitric oxide synthase
PRMT: protein arginine methyltransferase
Q-PCR: quantitative PCR
*RpL27*: ribosomal protein L27
*RpS11*: ribosomal protein S11
*SCAMP1*: secretory carrier membrane protein 1
SDMA: symmetric dimethylarginine
*Smad5*: SMA-related protein 5

## References

[pmed-0020264-b01] Vallance P, Leone A, Calver A, Collier J, Moncada S (1992). Accumulation of an endogenous inhibitor of nitric oxide synthesis in chronic renal failure. Lancet.

[pmed-0020264-b02] Achan V, Broadhead M, Malaki M, Whitley G, Leiper J (2003). Asymmetric dimethylarginine causes hypertension and cardiac dysfunction in humans and is actively metabolized by dimethylarginine dimethylaminohydrolase. Arterioscler Thromb Vasc Biol.

[pmed-0020264-b03] Cooke JP (2004). Asymmetrical dimethylarginine: The Uber marker?. Circulation.

[pmed-0020264-b04] Usui M, Matsuoka H, Miyazaki H, Ueda S, Okuda S (1998). Increased endogenous nitric oxide synthase inhibitor in patients with congestive heart failure. Life Sci.

[pmed-0020264-b05] Gorenflo M, Zheng C, Werle E, Fiehn W, Ulmer HE (2001). Plasma levels of asymmetrical dimethyl-L-arginine in patients with congenital heart disease and pulmonary hypertension. J Cardiovasc Pharmacol.

[pmed-0020264-b06] Boger RH, Bode-Boger SM, Szuba A, Tsao PS, Chan JR (1998). Asymmetric dimethylarginine (ADMA): A novel risk factor for endothelial dysfunction: Its role in hypercholesterolemia. Circulation.

[pmed-0020264-b07] Zoccali C, Bode-Boger S, Mallamaci F, Benedetto F, Tripepi G (2001). Plasma concentration of asymmetrical dimethylarginine and mortality in patients with end-stage renal disease: A prospective study. Lancet.

[pmed-0020264-b08] Valkonen VP, Paiva H, Salonen JT, Lakka TA, Lehtimaki T (2001). Risk of acute coronary events and serum concentration of asymmetrical dimethylarginine. Lancet.

[pmed-0020264-b09] Savvidou MD, Hingorani AD, Tsikas D, Frolich JC, Vallance P (2003). Endothelial dysfunction and raised plasma concentrations of asymmetric dimethylarginine in pregnant women who subsequently develop pre-eclampsia. Lancet.

[pmed-0020264-b10] Tsikas D, Boger RH, Sandmann J, Bode-Boger SM, Frolich JC (2000). Endogenous nitric oxide synthase inhibitors are responsible for the L-arginine paradox. FEBS Lett.

[pmed-0020264-b11] Chambers RC, Leoni P, Kaminski N, Laurent GJ, Heller RA (2003). Global expression profiling of fibroblast responses to transforming growth factor-beta1 reveals the induction of inhibitor of differentiation-1 and provides evidence of smooth muscle cell phenotypic switching. Am J Pathol.

[pmed-0020264-b12] Seo J, Bakay M, Chen YW, Hilmer S, Shneiderman B (2004). Interactively optimizing signal-to-noise ratios in expression profiling: Project-specific algorithm selection and detection *p*-value weighting in Affymetrix microarrays. Bioinformatics.

[pmed-0020264-b13] Teerlink T, Nijveldt RJ, de Jong S, van Leeuwen PA (2002). Determination of arginine, asymmetric dimethylarginine, and symmetric dimethylarginine in human plasma and other biological samples by high-performance liquid chromatography. Anal Biochem.

[pmed-0020264-b14] Dennis G, Sherman BT, Hosack DA, Yang J, Gao W (2003). DAVID: Database for Annotation, Visualization, and Integrated Discovery. Genome Biol.

[pmed-0020264-b15] Hosack DA, Glynn Dennis G, Sherman BT, Lane BC, Lempicki RA (2003). Identifying biological themes within lists of genes with EASE. Genome Biol.

[pmed-0020264-b16] Closs EI, Basha FZ, Habermeier A, Forstermann U (1997). Interference of L-arginine analogues with L-arginine transport mediated by the y+ carrier hCAT-2B. Nitric Oxide.

[pmed-0020264-b17] Tang J, Gary JD, Clarke S, Herschman HR (1998). PRMT 3, a type I protein arginine N-methyltransferase that differs from PRMT1 in its oligomerization, subcellular localization, substrate specificity, and regulation. J Biol Chem.

[pmed-0020264-b18] Kielstein JT, Bode-Boger SM, Frolich JC, Haller H, Boger RH (2001). Relationship of asymmetric dimethylarginine to dialysis treatment and atherosclerotic disease. Kidney Int Suppl.

[pmed-0020264-b19] Zoccali C (2002). Endothelial damage, asymmetric dimethylarginine and cardiovascular risk in end-stage renal disease. Blood Purif.

[pmed-0020264-b20] Stuhlinger MC, Abbasi F, Chu JW, Lamendola C, McLaughlin TL (2002). Relationship between insulin resistance and an endogenous nitric oxide synthase inhibitor. JAMA.

[pmed-0020264-b21] MacAllister RJ, Whitley GS, Vallance P (1994). Effects of guanidino and uremic compounds on nitric oxide pathways. Kidney Int.

[pmed-0020264-b22] Faruqi TR, Erzurum SC, Kaneko FT, DiCorleto PE (1997). Role of nitric oxide in poly(I-C)-induced endothelial cell expression of leukocyte adhesion molecules. Am J Physiol.

[pmed-0020264-b23] Hemish J, Nakaya N, Mittal V, Enikolopov G (2003). Nitric oxide activates diverse signaling pathways to regulate gene expression. J Biol Chem.

[pmed-0020264-b24] Cardounel AJ, Xia Y, Zweier JL (2005). Endogenous methylarginines modulate superoxide as well as nitric oxide generation from neuronal nitric oxide synthase: Differences in the effects of monomethyl and dimethyl arginines in the presence and absence of tetrahydrobiopterin. J Biol Chem.

[pmed-0020264-b25] Suda O, Tsutsui M, Morishita T, Tasaki H, Ueno S (2004). Asymmetric dimethylarginine causes arteriosclerotic lesions in endothelial nitric oxide synthase-deficient mice: Involvement of renin-angiotensin system and oxidative stress. Arterioscler Thromb Vasc Biol.

[pmed-0020264-b26] Smirnova IV, Kajstura M, Sawamura T, Goligorsky MS (2004). Asymmetric dimethylarginine upregulates LOX-1 in activated macrophages: Role in foam cell formation. Am J Physiol Heart Circ Physiol.

[pmed-0020264-b27] Cross JM, Donald AE, Kharbanda R, Deanfield JE, Woolfson RG (2001). Acute administration of L-arginine does not improve arterial endothelial function in chronic renal failure. Kidney Int.

[pmed-0020264-b28] Arrigoni FI, Vallance P, Haworth SG, Leiper JM (2003). Metabolism of asymmetric dimethylarginines is regulated in the lung developmentally and with pulmonary hypertension induced by hypobaric hypoxia. Circulation.

[pmed-0020264-b29] Millatt LJ, Whitley GS, Li D, Leiper JM, Siragy HM (2003). Evidence for dysregulation of dimethylarginine dimethylaminohydrolase I in chronic hypoxia-induced pulmonary hypertension. Circulation.

[pmed-0020264-b30] Deng Z, Morse JH, Slager SL, Cuervo N, Moore KJ (2000). Familial primary pulmonary hypertension (gene *PPH1*) is caused by mutations in the bone morphogenetic protein receptor-II gene. Am J Hum Genet.

[pmed-0020264-b31] Hruska KA, Saab G, Chaudhary LR, Quinn CO, Lund RJ (2004). Kidney-bone, bone-kidney, and cell–cell communications in renal osteodystrophy. Semin Nephrol.

[pmed-0020264-b32] Xiao ZS, Quarles LD, Chen QQ, Yu YH, Qu XP (2001). Effect of asymmetric dimethylarginine on osteoblastic differentiation. Kidney Int.

[pmed-0020264-b33] Kearns AE, Donohue MM, Sanyal B, Demay MB (2001). Cloning and characterization of a novel protein kinase that impairs osteoblast differentiation in vitro. J Biol Chem.

[pmed-0020264-b34] Boger RH, Sydow K, Borlak J, Thum T, Lenzen H (2000). LDL cholesterol upregulates synthesis of asymmetrical dimethylarginine in human endothelial cells: Involvement of S-adenosylmethionine-dependent methyltransferases. Circ Res.

[pmed-0020264-b35] Bachand F, Silver PA (2004). PRMT3 is a ribosomal protein methyltransferase that affects the cellular levels of ribosomal subunits. EMBO J.

[pmed-0020264-b36] Casellas P, Jeanteur P (1978). Protein methylation in animal cells. II. Inhibition of S-adenosyl-L-methionine:protein(arginine) N-methyltransferase by analogs of S-adenosyl-L-homocysteine. Biochim Biophys Acta.

